# Adolescent Cardiovascular Risk Trajectories and Later-Life Maternal Morbidity

**DOI:** 10.1001/jamanetworkopen.2025.36095

**Published:** 2025-10-10

**Authors:** Katharine J. McCarthy, Annabelle Ng, Natalie A. Boychuk, Teresa Janevic

**Affiliations:** 1 Department of Population Health Science and Policy, Icahn School of Medicine at Mount Sinai, New York, New York; 2 Department of Obstetrics, Gynecology, and Reproductive Science, Icahn School of Medicine at Mount Sinai, New York, New York; 3 Department of Epidemiology, Mailman School of Public Health, Columbia University, New York, New York

## Abstract

**Importance:**

There is an increasing burden of cardiovascular-related morbidity in pregnancy. To avert excess cardiovascular health (CVH) risks, a clearer understanding of the association between adolescent and young-adult cardiovascular risks and future pregnancy health is warranted.

**Objective:**

To assess the association of cardiovascular risk in adolescence and young adulthood with later-life adverse pregnancy outcomes.

**Design, Setting, and Participants:**

This cohort study used data from the National Longitudinal Study of Adolescent and Adult Health. Female participants interviewed in 1994-1996 (ages 11-19 years), 2001-2002 (ages 18-26 years), and 2016-2018 (ages 34-43 years) without juvenile-onset diabetes or hypertension and with reported birth data were included in the sample.

**Exposure:**

CVH risk in adolescence and young adulthood was categorized as low, medium, or high using an adapted Life’s Essential 8 scale (range: 0-100, with a score of 0 representing poor CVH status and 100 indicating optimal CVH status).

**Main Outcomes and Measures:**

The primary outcome was a self-reported history of gestational diabetes (GD) or hypertensive disorder of pregnancy (HDP) between survey waves 3 and 5. Associations between adolescent CVH and future GD or HDP were estimated using log-binomial regression, adjusting for maternal characteristics and sociodemographic factors. Analyses accounted for the sampling design by controlling for clustered data and unequal probability of selection and were weighted to represent population-level estimates.

**Results:**

The sample included 1094 female participants (median [IQR] age, 16 [14-17] years) with a median (IQR) body mass index (calculated as weight in kilograms divided by height in meters squared) of 21.6 (19.6-24.5). Participants with poor adolescent CVH compared with optimal adolescent CVH had an adjusted risk ratio (ARR) of 2.01 (95% CI, 0.96-4.24) for a future elevated risk of GD. The ARR for future GD for intermediate (vs optimal) CVH was 1.04 (95% CI, 0.53-2.07). The highest incidence of GD was observed among participants with poor CVH during adolescence and young adulthood (28.5%) compared with an incidence of 9.6% among those with intermediate CVH and 4.7% among those with optimal CVH during adolescence and young adulthood. Participants with poor adolescent CVH who had optimal or intermediate CVH in young adulthood had a 24.9% and 14.7% incidence of GD, respectively. Compared with optimal CVH status, poor adolescent CVH had an ARR of 1.22 (95% CI, 0.74-2.01), and intermediate adolescent CVH had an ARR of 1.43 (95% CI, 0.93-2.21) for a future elevated risk of HDP.

**Conclusions and Relevance:**

In this cohort study, the incidence of GD was higher among those with poor CVH in adolescence and young adulthood. Achieving optimal CVH in adolescence may reduce the risk of GD in adulthood.

## Key Points

**Question:**

Is cardiovascular health (CVH) status in adolescence and young adulthood associated with adverse pregnancy outcomes in later life?

**Findings:**

In this cohort study of 1094 participants, optimal CVH (defined by a Life’s Essential 8 risk score ranging from 80 to 100) in adolescence and young adulthood vs poor and intermediate CVH had lower risk estimates for gestational diabetes and hypertensive disorders of pregnancy in adulthood. The highest incidence of gestational diabetes was observed among participants with poor CVH in adolescence and young adulthood.

**Meaning:**

Optimal CVH in adolescence may be key in preventing cardiovascular-related morbidity in pregnancy and later life.

## Teaser

This cohort study assesses cardiovascular health in adolescence and the future risk of gestational diabetes and hypertensive disorders of pregnancy.

## Introduction

Cardiovascular disease is the primary cause of maternal morbidity and mortality in the US, accounting for nearly one-third of maternal deaths from 2017 to 2019.^1^ Pregnancy may also unmask existing latent disease processes that do not appear again until later in adulthood, serving as an early warning system.^2,3,4^ Pregnancy complications, including gestational diabetes (GD) and hypertensive disorders of pregnancy (HDP), have implications for future cardiovascular health (CVH) and have been associated with elevated risk of later-life maternal type 2 diabetes, hypertension, stroke, and myocardial infarction.^2,4,5^ GD and HDP may have lifelong implications for child development and have been associated with adverse newborn outcomes, including stillbirth,^5^ preterm delivery,^6,7^ newborn intensive care unit admission,^8^^,^ infant macrosomia, and low birth weight.^9,10^ Considering that prepregnancy risk factors such as obesity, diabetes, and chronic hypertension have steadily increased over the past 2 decades,^11^ without appropriate intervention, the burden of adverse maternal and newborn outcomes may increase.

Prenatal CVH interventions largely fall into 2 types: those targeting lifestyle behaviors (eg, diet and exercise) and those using pharmacological interventions (eg, insulin-sensitizing drugs) during pregnancy.^12^ Pregnancy-initiated interventions, however, have limited scalability and may not be sufficient to reduce transmission of intergenerational risk factors.^13,14^ Approaches targeting the preconception phase are hindered by the fact that an estimated 47% of pregnancies are unintended or mistimed at the time of conception,^15^ and many women do not access care prior to pregnancy due to limited access, delayed recognition, or lack of engagement with the health care system.^16^ Considering the adverse implications of unhealthy behavioral patterns over time and that sufficient time is needed to achieve optimal health, adolescence and young adulthood may be critical phases in the life course continuum.^12,17^

Adolescence and young adulthood are critical times of developmental plasticity,^18^ where behavioral patterns, including smoking, dietary, and exercise habits, are often established and solidified. These behaviors can have cumulative implications for adult health and carry intergenerational risks. For example, higher nutrition knowledge and healthier dietary behaviors in adolescence have been associated with lower cardiovascular risk in adulthood,^19^ while sustained improvements in CVH during young adulthood are associated with reduced long-term morbidity.^20^ Additionally, poor nutrition in pregnancy has been associated with increased obesity in offspring.^21^ Late adolescence and young adulthood represent a critical inflection point where loss of CVH status begins to accelerate into midlife.^20,22,23^ Pregnancy is a more proximal window into latent cardiovascular risk trajectories; however, most prior research has examined the implications of pregnancy complications for postpartum CVH^2,24^ or obesity among offspring.^25,26,27^ This study investigates the hypothesis that CVH in adolescence and young adulthood is associated with later-life adverse pregnancy outcomes.

## Methods

### Data Source

This cohort study used data from the National Longitudinal Study of Adolescent and Adult Health (or Add Health), a nationally representative longitudinal cohort of in-school adolescents that seeks to better understand developmental trajectories of adolescent health into adulthood.^2,3,4^ We included data from waves 1 (1994-1995; participants aged 11-18 years and their parents), 2 (1996; participants aged 12-19 years), 3 (2001-2002; participants aged 18-26 years), 4 (2007-2008; participants aged 24-32 years), and 5 (2016-2018, participants aged 33-43 years). This study was deemed exempt by the institutional review board at the Icahn School of Medicine at Mount Sinai, with a waiver of informed consent because it involved secondary analysis of deidentified data. The reporting adheres to the Strengthening the Reporting of Observational Studies in Epidemiology (STROBE) guideline.^28^

### Sample

Our analysis included a subset of the original 10 480 female participants interviewed at baseline (wave 1) with wave 1 parental survey data. We included participants who were reinterviewed in waves 2 (ages 12-19 years) through 5 and those who ever gave birth between waves 3 to 5 (ages 20-43 years) (eFigure 1 in Supplement 1). We excluded 281 individuals who gave birth prior to wave 3 and who had pregestational diabetes or preexisting hypertension. Additionally, 776 participants with missing data on both outcomes, CVH risk indicators, and covariates were excluded.

### Measures

#### Outcome

Main outcomes were self-reported history of ever experiencing (1) GD or (2) HDP between waves 3 and 5. GD was ascertained from the question, “Have you ever experienced diabetes during a pregnancy?” (yes or no); this question has shown high sensitivity and specificity via self-report.^29^ A combined indicator for any HDP (yes or no) was created by report of any of the following complications: ever having high blood pressure or hypertension; preeclampsia or toxemia; protein in urine; or seizures, convulsions, or eclampsia during a pregnancy. As secondary outcomes, we disaggregated HDP into separate indicators for gestational hypertension (ie, whether participants reported ever having high blood pressure or hypertension during a pregnancy) and preeclampsia or eclampsia (ever having preeclampsia, toxemia, seizures, convulsions or eclampsia during a pregnancy). Prior validation studies of self-reported HDP have found moderate to high positive predictive values, with false-positive results declining with age.^28,30^

#### Exposure

Our exposure of interest was CVH status in adolescence and young adulthood. We calculated CVH using an adapted version of the American Heart Association (AHA) Life’s Essential 8 (LE8) scale. For the adolescent measure, we used the scoring system for individuals aged 19 years or younger.^31^ Indicators available in the Add Health dataset at waves 2 and 3 included self-reported diet, physical activity, nicotine exposure, sleep health, and body mass index (BMI; calculated as weight in kilograms divided by height in meters squared). Information on blood lipid levels, blood glucose levels, and blood pressure was not available at the time points measured and was not included. The continuous risk score was the mean of the component metric scores ranging from 0 to 100, with a score of 0 representing poor CVH and 100 indicating optimal CVH. Risk was categorized as high (score, 0-49), intermediate (score, 50-79), and low (score, 80-100), informed by AHA guidelines.^31^ Detailed information on the CVH measures and scoring is presented in eTable 1 in Supplement 1.

#### Covariates

Covariates were self-reported and included age in years (estimated using birth month and year), race and ethnicity, nativity (US or other country), parental education, insurance status, and wave of first birth. Birth date, race and ethnicity, and nativity were collected in the wave 1 in-home survey. We consider race and ethnicity as proxies for experiences of racism, which could influence social determinants of health and confound the relationship between adolescent CVH and hypertension or diabetes during pregnancy. We categorized race and ethnicity as Asian, non-Hispanic (hereafter Asian); Black, non-Hispanic (hereafter Black); Hispanic, all races; and White, non-Hispanic (hereafter White). Due to data sparsity, we created a composite category for multiracial and/or other race (American Indian or Native American or race not otherwise specified). Parental education was self-reported in a separate survey of parents of wave-1 respondents and was categorized as less than a high school diploma, high school diploma, some college, and college degree or higher. Insurance status in young adulthood (wave 3) was categorized as public, private, or no insurance. Finally, date of first birth was ascertained in waves 4 and 5.

### Statistical Analysis

Statistical analysis was performed between July 2024 and January 2025 using R, version 4.4.1 (R Project for Statistical Computing). For all analyses, we used a complete case analysis approach, excluding participants with missing data on CVH risk indicators, pregnancy outcomes, and covariates. We used log-binomial regression to calculate the adjusted relative risk (ARR) of GD and HDP by adolescent CVH. Adjusted models controlled for maternal characteristics and sociodemographic factors, which were hypothesized confounders according to our directed acyclic graph (eFigure 2 in Supplement 1). Parental education and insurance status were included as proxies for participant socioeconomic status. We did not adjust for country of birth due to minimal variation in the sample. We calculated the cumulative incidence of GD and HDP among participants by their CVH status in adolescence and young adulthood to gain insight into the role of transitions in CVH over time. Finally, we estimated the degree to which the burden of GD and HDP could be prevented if CVH was optimized in adolescence by calculating the population attributable risk (PAR) using the following formula: PAR = *p* × (ARR – 1) / 1 + *p* × (ARR – 1), where *p* represents the prevalence of poor or intermediate adolescent CVH status and the ARR is the risk of GD or HDP. We then summed the PAR for poor and intermediate CVH. Analyses accounted for the sampling design by controlling for clustered data and unequal probability of selection and are weighted to represent population-average estimates. In the sensitivity analysis, outcomes, exposures, and covariates were compared between the complete case analysis sample and the larger birth sample to address potential bias from excluding participants with missing data (eTable 2 in Supplement 1).

## Results

The complete case analysis sample included 1094 female participants (median [IQR] age 16 [14-17] years) who were first interviewed during adolescence with data on either GD or HDP. Of these individuals, 1.3% self-identified as Asian, 13.6% as Black, 8.0% as Hispanic, 70.4% as White, and 6.7% as other race or ethnicity. Most participants (96.9%) were born in the US, had parents with a high school diploma or less (53.4%), and had private insurance (62.8%). Most participants (63.1%) were characterized as having intermediate CVH, followed by those with optimal (18.9%) and poor CVH (18.0%) in adolescence (Table 1). By young adulthood, the prevalence of optimal CVH increased to 22.8%, while the prevalence of intermediate CVH or poor CVH decreased to 59.5% and 17.7%, respectively. The prevalence of optimal CVH was highest among Hispanic participants (35.5%), followed by White (18.4%) and Black (15.1%) participants. In contrast, 19.2% of White, 14.7% of Black, and 14.6% of Hispanic participants had poor CVH. Those participants with poor CVH were less likely to have parents with educational attainment higher than a high school diploma and were more likely to have public or no insurance in adulthood. We found few differences between the larger birthing sample and the complete case analysis sample (eTable 2 in Supplement 1).

**Table 1.** Sociodemographic Characteristics of the Add Health Birth Cohort by Prepregnancy Cardiovascular Health (CVH) Status^a^

Characteristic	Total (n = 1094)	Individuals by CVH risk, unweighted No./total No. (weighted %)^b^		
		Optimal (n = 220)	Intermediate (n = 679)	Poor (n = 195)
Age, median (IQR), y^c^	16 (14-17)	15 (14-17)	16 (14-17)	17 (15-17)
Age group, y^c^				
12-14	204 (25.6)	59/204 (30.7)	129/204 (62.6)	16/204 (6.7)
15-17	689 (59.4)	124/689 (14.6)	426/689 (63.8)	139/689 (21.5)
18-22	201 (15.0)	37/201 (15.7)	124/201 (61.1)	40/201 (23.2)
BMI, median (IQR)^c^	21.6 (19.6-24.5)	21.1 (19.2-22.9)	21.5 (19.7-24.7)	22.6 (19.6-28.7)
Race and ethnicity^c^				
Asian	31 (1.3)	NR^d^	NR^d^	NR^d^
Black	200 (13.6)	34/200 (15.1)	134 /200 (70.2)	32/200 (14.7)
Hispanic	123 (8.0)	32/123 (35.5)	72/123 (49.9)	19/123 (14.6)
White	664 (70.4)	140/664 (18.4)	395/664 (62.5)	129/664 (19.2)
Other^e^	76 (6.7)	NR^d^	NR^d^	NR^d^
Nativity^c^				
US	1045 (96.9)	210/1045 (18.7)	645/1045(63.1)	190/1045(18.3)
Other country	49 (3.1)	NR^d^	NR^d^	NR^d^
Parental education^f^				
<High school diploma	169 (15.6)	27/169 (14.7)	113169 (68.9)	29/169 (16.4)
High school diploma	375 (37.8)	77/375 (20.8)	220/375 (57.3)	78/375 (21.8)
Some college	334 (30.0)	69/334 (18.1)	208/334 (66.9)	57/334 (15.0)
College degree or higher	216 (16.6)	47/216 (20.0)	138/216 (63.7)	31/216 (16.3)
Insurance^g^				
Medicaid or none	352 (37.2)	55/352 (16.9)	220/352 (61.7)	77/352 (21.4)
Private insurance	742 (62.8)	165/742 (20.1)	459/742 (63.9)	118/742 (16.0)
Wave of first birth, median (IQR)	3 (3-4)	4 (3-4)	3 (3-4)	3 (3-4)
HDP^h^				
No	825 (75.5)	173/825 (20.2)	507/825 (61.5)	145/825 (18.3)
Yes	269 (24.5)	47/269 (14.9)	172/269 (68.1)	50/269 (17.0)
Gestational hypertension^h^				
No	882 (81.1)	181/882 (19.3)	544/882 (62.6)	157/882(18.1)
Yes	209 (18.5)	39/209 (17.5)	132/209 (64.7)	38/209 (17.8)
Missing data	3 (0.3)	0/3	3/3	0/3
Preeclampsia or eclampsia^h^				
No	915 (83.2)	191/915 (20.0)	561/915 (61.1)	163/915 (18.9)
Yes	176 (16.5)	29/176 (13.5)	115/176 (72.7)	32/176 (13.8)
Missing data	3 (0.3)	0/3	3/3	0/3
GD^h^				
No	969 (90.4)	202/969 (19.4)	610/969(64.0)	157/969 (16.6)
Yes	121 (9.3)	18/121 (14.9)	66/121 (54.1)	37/121 (31.0)
Missing data	4 (0.2)	0/4	3/4	1/4

Abbreviations: BMI, body mass index (calculated as weight in kilograms divided by height in meters squared); CVH, cardiovascular health; HDP, hypertensive disorders of pregnancy; GD, gestational diabetes; NR, not reported.

^a^ Participants with no history of prepregnancy diabetes or preexisting hypertension with data on either GD or HDP.

^b^ CVH risk score adapted from Life’s Essential 8 for adolescents measured at the National Longitudinal Study of Adolescent and Adult Health wave 2 (ages 12-22) using the following cutoff scores: 80 to 100 (optimal CVH status), 50 to 79 (intermediate CVH status), and 0 to 49 (poor CVH status).

^c^ Measured at wave 2 (adolescence).

^d^ Estimates were not reported due to small cell counts (≤10), as recommended by the National Longitudinal Study of Adolescent and Adult Health guidelines.

^e^ Other included American Indian or Native American, multiracial, and any race not otherwise specified.

^f^ Measured in wave 1 (adolescence).

^g^ Measured at wave 3 (young adulthood).

^h^ Measured at wave 5 (adulthood).

### ARR of GD and HDP in Adulthood by Adolescent CVH Status

Among participants with GD, 31.0% had poor, 54.1% had intermediate, and 14.9% had optimal adolescent CVH. HDP was present in 24.5% of participants, of which 17.0% had poor CVH compared with 68.1% with intermediate and 14.9% with optimal CVH status (Table 1). Adjusting for covariates, we found that the participants with poor adolescent CVH compared with those with optimal CVH had an ARR for future GD of 2.01 (95% CI, 0.96-4.24) (Table 2). Participants with intermediate adolescent CVH had no increased risk of GD relative to those with optimal CVH (ARR, 1.04; 95% CI, 0.53-2.07). There was no statistically significant increased risk of HDP in later pregnancy among participants with poor (ARR, 1.22; 95% CI, 0.74-2.01) or intermediate (ARR, 1.43; 95% CI, 0.93-2.21) adolescent CVH compared with those with optimal CVH. When the model was restricted to the preeclampsia or eclampsia pregnancy outcome, participants with intermediate vs optimal adolescent CVH had an ARR of 1.63 (95% CI, 0.96-2.76) (Table 2). No association between any level of adolescent CVH and risk of gestational hypertension was observed when the pregnancy outcome was narrowed to gestational hypertension.

**Table 2.** Adjusted Relative Risk of GD and Adverse Birth Outcomes by Prepregnancy CVH Status Estimated Using Log-Binomial Regression and Sampling Weights^a^

Outcome	Adolescent CVH status		
	Optimal	Intermediate	Poor
GD			
No./total No.	18/220	66/677	37/194
RR	1 [Reference]	1.09 (0.57-2.08)	2.19 (1.11-4.31)
ARR (95% CI)	1 [Reference]	1.04 (0.53-2.07)	2.01 (0.96-4.24)
HDP			
No./total No.	47/220	173/680	50/195
RR	1 [Reference]	1.37 (0.89-2.11)	1.20 (0.72-2.01)
ARR (95% CI)	1 [Reference]	1.43 (0.93-2.21)	1.22 (0.74-2.01)
Gestational hypertension			
No./total No.	39/220	133/677	38/195
RR	1 [Reference]	1.11 (0.69-1.80)	1.07 (0.58-1.96)
ARR (95% CI)	1 [Reference]	1.19 (0.74-1.90)	1.13 (0.64-2.00)
Preeclampsia or eclampsia			
No./total No.	28/217	117/681	32/198
RR	1 [Reference]	1.62 (0.92-2.82)	1.07 (0.57-2.02)
ARR (95% CI)	1 [Reference]	1.63 (0.96-2.76)	1.03 (0.56-1.87)

Abbreviations: ARR, adjusted relative risk; CVH, cardiovascular health; GD, gestational diabetes; HDP, hypertensive disorders of pregnancy; RR, relative risk.

^a^ Adjusted for age, race or ethnicity, parental education, insurance status, wave at first birth.

### Cumulative Incidence of GD and HDP by Transition in CVH Risk Status Between Adolescence and Young Adulthood

Among birthing individuals with CVH measures in adolescence and young adulthood, we found that the highest incidence of GD was among those who retained poor CVH status (28.5%) relative to those who maintained optimal (4.7%) or intermediate CVH status (9.6%) (Table 3). Compared with participants who retained poor CVH status over time, the incidence of GD was lower among those whose CVH status changed from poor to optimal (24.9%) or from poor to intermediate (14.7%). With regard to HDP, those with optimal CVH in adolescence and adulthood had the lowest incidence (14.8%). However, participants with poor CVH in adolescence who achieved optimal status in young adulthood had the highest HDP incidence (33.6%).

**Table 3.** Incidence of Future GD and HDP Among Birthing Individuals by Cardiovascular Risk Trajectories From Adolescence to Young Adulthood

Transition in CVH status from adolescence to young adulthood	Individuals, unweighted No./total No. (weighted incidence, %)	
	GD	HDP
Optimal CVH status in adolescence		
No. with complete data for CVH status	180	206
No transition from optimal	6/70 (4.7)	11/69 (14.8)
Optimal to intermediate	9/99 (10.5)	20/99 (20.0)
Optimal to poor	2/11 (19.5)	4/11 (16.2)
Intermediate CVH status in adolescence		
No. with complete data for CVH status	554	557
No transition from intermediate	38/346 (9.6)	94/348 (28.8)
Intermediate to optimal	8/138 (3.1)	32/139 (21.8)
Intermediate to poor	5/70 (5.8)	19/70 (30.3)
Poor CVH status in adolescence		
No. with complete data for CVH status	148	149
No transition from poor	14/42 (28.5)	8/42 (20.9)
Poor to optimal	3/17 (24.9)	3/17 (33.6)
Poor to intermediate	16/89 (14.7)	28/90 (26.6)

Abbreviations: CVH, cardiovascular health; GD, gestational diabetes; HDP, hypertensive disorders of pregnancy.

### CVH Optimization in Adolescence and Future Risk of GD and HDP

We calculated the population attributable risk to assess the degree to which the burden of GD and HDP could be prevented if CVH was optimized in adolescence. We found that, overall, preventing poor or intermediate CVH status in adolescence could reduce GD risk from 9.3% to 7.6%, with a 15.9% risk reduction for preventing poor CVH and an additional 2.5% reduction for preventing intermediate CVH. Preventing poor or intermediate CVH in adolescence could reduce HDP risk from 24.5% to 18.3%, with 3.8% risk reduction attributable to poor CVH and an additional 21.3% for intermediate CVH, respectively.

## Discussion

In this school-based cohort of birthing individuals, we found no association between poor adolescent CVH and GD or HDP. Participants with optimal CVH in both adolescence and young adulthood had the lowest incidence of GD and HDP, and those with poor CVH status at both stages had the highest incidence of GD. Compared with HDP, we observed an ARR greater than 2 between our largely behavioral measure of CVH with GD, which may reflect differences in the underlying pathophysiology. HDP represents a heterogeneous group of conditions, including gestational hypertension, preeclampsia, and superimposed chronic hypertension, with distinct underlying mechanisms.^32^ GD is predominantly associated with insulin resistance and dysregulated glucose metabolism, which are modifiable through diet and physical activity.^33^ HDP is more commonly related to abnormal placentation, vascular endothelial dysfunction, and systemic inflammation.^34^ This divergence in underlying biology may partly explain why observed outcomes were less consistent for HDP than for GD. However, while mechanisms underlying pregnancy complications are intricate and likely influenced by multiple factors, our results nonetheless suggest that unhealthy BMI and behavioral patterns originating in adolescence and young adulthood are associated with later-life pregnancy complications.^17^ Earlier life phases may be critical opportunities to disrupt CVH risk trajectories by intervening on modifiable risk factors before the onset of later-life maternal morbidity. This study adds to scant evidence exploring the role of adolescent and young adult CVH in future maternal health outcomes. While our CVH measure focuses primarily on behavioral factors, including all health behaviors in AHA’s LE8 framework plus BMI, related research demonstrates how singular clinical measures of CVH are associated with later adverse pregnancy outcomes. Specifically, prospective evidence among individuals aged 10 to 24 years has shown that those with prepregnancy prediabetes measured using hemoglobin A_1c_, had more than twice the risk of GD at a future first birth.^35^ Other research has documented the association between adolescent and child BMI or body size with later GD.^36,37,38^ Our results support the clinical utility of the LE8 cardiovascular construct for birthing individuals and its importance as a clinical target across the prepregnancy lifespan.^17^

Beyond adolescence alone, the results illustrate how an individual’s trajectory of cardiovascular function across the prepregnancy period has implications for later pregnancy health. We found that participants who had poor CVH in adolescence continued to have poor status in young adulthood had the highest incidence of later GD (28.5%)—roughly 1.5 times the burden compared with those whose CVH status declined from optimal to poor between life stages (19.5%). In contrast, the lowest incidence of both GD and HDP was observed among participants whose CVH status remained optimal at both life stages. Taken together, these results suggest pregnancy-related risks may begin to accumulate in adolescence and young adulthood. However, our results are descriptive in nature, and replication in other longitudinal cohorts is warranted to better understand the contribution that timing and duration of poor preconception CVH have for future maternal health risks.

While less explored in relation to maternal morbidity, accumulation of CVH risk with longer duration of exposure (accumulation hypothesis), has been well documented with respect to cardiovascular disease in middle age and beyond.^39,40,41^ The long-term outcomes of elevated blood pressure, high cholesterol, and tobacco smoke exposure in childhood have been shown to be greatly reduced if exposures are mitigated or removed by adulthood.^40^ For example, evidence has demonstrated that initiation of cholesterol-lowering agents in childhood reduces adult CVH risk more than starting these agents in adulthood.^42^ Similar outcomes have been documented with regard to smoking cessation timing and lung cancer risk.^43^ Our estimates of population attributable risk similarly suggest that CVH risk mitigation earlier in the life course can reduce excess maternal health risks. Interventions supporting early-life CVH optimization from adolescence to adulthood, such as maintaining an active lifestyle,^44^ nutritional knowledge, and healthy diet,^19^ have shown promise in mitigating CVH risk in adulthood. These findings point to the need for additional prospective research to address questions, such as whether greater reductions in maternal CVH-related morbidity might be achieved by interventions beginning in childhood and adolescence than those targeted to pregnancy.

### Strengths and Limitations

Our study has several strengths, including the use of a large prospective cohort, which allows longitudinal assessment of CVH status over time. Our measure of adult CVH included 5 behavioral factors and BMI. However, several limitations warrant mention. First, not all clinical risk factors of CVH included in the LE8 framework (blood pressure, blood lipid level, and blood glucose level) were available before adulthood. Our measure of CVH status in adolescence and young adulthood includes CVH-related health behaviors as well as BMI. It is also possible that our prospective CVH measures do not completely capture fluctuation in status due to lifestyle changes between data collection time points. Second, our measure of pregnancy complications relied on participants’ retrospective self-reports rather than medical records. Self-reported diabetes during pregnancy has shown greater than 90% sensitivity and specificity while self-reported HDP showed moderate sensitivity (77%) and high specificity (96%).^45^ Last, due to sample size constraints, we provided descriptive results to show the percentage of individuals who either maintained or changed their CVH status over time and the cumulative incidence of GD or HDP. Small sample sizes in some comparison groups may also have reduced statistical comparisons for HDP and its subtypes. This study’s data were weighted to represent national estimates; however, analyses should be replicated in other cohorts to improve generalizability given the sample size. Due to missing covariate data, we also chose to control for insurance status in young adulthood only and did not account for changes in insurance status between waves 3 to 5. As in all observational cohort studies, there is the potential for residual confounding due to unmeasured variables or reporting bias, which may contribute to biased estimated outcomes.

## Conclusions

In this cohort study, we found that individuals with poor CVH in adolescence and young adulthood had a higher incidence of GD in adulthood than those with intermediate or optimal CVH. The CVH measure was defined using all of the behavioral factors in the AHA’s LE8 in addition to BMI. Considering the increasing prevalence of risk factors including obesity, prediabetes, and hypertension among adolescents, investing in adolescent health may benefit life course and intergenerational health.

## Notes

Supplement 1. **eFigure 1.** Study flowchart **eTable 1.** Constructed CVH risk score measures in Waves II and III **eTable 2.** Unweighted counts and weighted frequencies of samples with and without complete data for preconception cardiovascular risk score, age, race/ethnicity, nativity, parental education, insurance status, and wave of first birth, among women without preexisting diabetes and hypertension who gave birth sometime between Waves III to V (birthing sample) **eFigure 2.** Directed acyclic graph of relationship between adolescent cardiovascular health status (CVH) and later life adverse pregnancy outcomes (APO)
eReference

Supplement 2.
Data Sharing Statement

## References

[1] Petersen EE, Davis NL, Goodman D, . Vital signs: pregnancy-related deaths, United States, 2011-2015, and strategies for prevention, 13 states, 2013-2017. MMWR Morb Mortal Wkly Rep. 2019;68(18):423-429. doi:10.15585/mmwr.mm6818e1 31071074 PMC6542194

[2] Staff AC, Redman CWG, Williams D, ; Global Pregnancy Collaboration (CoLab). Pregnancy and long-term maternal cardiovascular health: progress through harmonization of research cohorts and biobanks. Hypertension. 2016;67(2):251-260. doi:10.1161/HYPERTENSIONAHA.115.06357 26667417

[3] Poon LC, Nguyen-Hoang L, Smith GN, ; FIGO Committee on Impact of Pregnancy on Long-term Health and the FIGO Division of Maternal and Newborn Health. Hypertensive disorders of pregnancy and long-term cardiovascular health: FIGO Best Practice Advice. Int J Gynaecol Obstet. 2023;160(S1)(suppl 1):22-34. doi:10.1002/ijgo.14540 36635079

[4] McCarthy KJ, Liu SH, Huynh M, . Influence of gestational diabetes mellitus on diabetes risk and glycemic control in a retrospective population-based cohort. Diabetes Care. 2023;46(8):1483-1491. doi:10.2337/dc22-1676 37341505 PMC10369124

[5] Grandi SM, Filion KB, Yoon S, . Cardiovascular disease-related morbidity and mortality in women with a history of pregnancy complications. Circulation. 2019;139(8):1069-1079. doi:10.1161/CIRCULATIONAHA.118.036748 30779636

[6] Tanz LJ, Stuart JJ, Williams PL, . Preterm delivery and maternal cardiovascular disease risk factors: the nurses’ health study II. J Womens Health (Larchmt). 2019;28(5):677-685. doi:10.1089/jwh.2018.7150 30222501 PMC6537114

[7] Heida KY, Bots ML, de Groot CJM, . Cardiovascular risk management after reproductive and pregnancy-related disorders: a Dutch multidisciplinary evidence-based guideline. Eur J Prev Cardiol. 2016;23(17):1863-1879. doi:10.1177/2047487316659573 27432836

[8] Vats H, Saxena R, Sachdeva MP, Walia GK, Gupta V. Impact of maternal pre-pregnancy body mass index on maternal, fetal and neonatal adverse outcomes in the worldwide populations: A systematic review and meta-analysis. Obes Res Clin Pract. 2021;15(6):536-545. doi:10.1016/j.orcp.2021.10.005 34782256

[9] Kc K, Shakya S, Zhang H. Gestational diabetes mellitus and macrosomia: a literature review. Ann Nutr Metab. 2015;66(suppl 2):14-20. doi:10.1159/000371628 26045324

[10] Morken NH, Halland F, DeRoo LA, Wilcox AJ, Skjaerven R. Offspring birthweight by gestational age and parental cardiovascular mortality: a population-based cohort study. BJOG. 2018;125(3):336-341. doi:10.1111/1471-0528.14522 28165208 PMC5821431

[11] Bhupathiraju SN, Hu FB. Epidemiology of obesity and diabetes and their cardiovascular complications. Circ Res. 2016;118(11):1723-1735. doi:10.1161/CIRCRESAHA.115.306825 27230638 PMC4887150

[12] Stephenson J, Heslehurst N, Hall J, . Before the beginning: nutrition and lifestyle in the preconception period and its importance for future health. Lancet. 2018;391(10132):1830-1841. doi:10.1016/S0140-6736(18)30311-8 29673873 PMC6075697

[13] Rogozińska E, Marlin N, Jackson L, . Effects of antenatal diet and physical activity on maternal and fetal outcomes: individual patient data meta-analysis and health economic evaluation. Health Technol Assess. 2017;21(41):1-158. doi:10.3310/hta21410 28795682 PMC5572115

[14] Syngelaki A, Nicolaides KH, Balani J, . Metformin versus placebo in obese pregnant women without diabetes mellitus. N Engl J Med. 2016;374(5):434-443. doi:10.1056/NEJMoa1509819 26840133

[15] Bearak JM, Popinchalk A, Beavin C, . Country-specific estimates of unintended pregnancy and abortion incidence: a global comparative analysis of levels in 2015-2019. BMJ Glob Health. 2022;7(3):e007151. doi:10.1136/bmjgh-2021-007151 35332057 PMC8943721

[16] Kaiser Family Foundation. Women’s coverage, access, and affordability: key findings from the 2017 Kaiser women’s health survey. March 13, 2018. Accessed May 18, 2022. https://www.kff.org/womens-health-policy/issue-brief/womens-coverage-access-and-affordability-key-findings-from-the-2017-kaiser-womens-health-survey/

[17] Khan SS, Brewer LC, Canobbio MM, ; American Heart Association Council on Epidemiology and Prevention; Council on Clinical Cardiology; Council on Cardiovascular and Stroke Nursing; Council on Arteriosclerosis, Thrombosis and Vascular Biology; Council on Hypertension; Council on Lifestyle and Cardiometabolic Health; Council on Peripheral Vascular Disease; and Stroke Council. Optimizing prepregnancy cardiovascular health to improve outcomes in pregnant and postpartum individuals and offspring: a scientific statement from the American Heart Association. Circulation. 2023;147(7):e76-e91. doi:10.1161/CIR.0000000000001124 36780391 PMC10080475

[18] Hochberg Z, Belsky J. Evo-devo of human adolescence: beyond disease models of early puberty. BMC Med. 2013;11(1):113. doi:10.1186/1741-7015-11-113 23627891 PMC3639027

[19] Morcel J, Béghin L, Michels N, . Nutritional and physical fitness parameters in adolescence impact cardiovascular health in adulthood. Clin Nutr. 2024;43(8):1857-1864. doi:10.1016/j.clnu.2024.06.022 38959665

[20] Allen NB, Krefman AE, Labarthe D, . Cardiovascular health trajectories from childhood through middle age and their association with subclinical atherosclerosis. JAMA Cardiol. 2020;5(5):557-566. doi:10.1001/jamacardio.2020.0140 32159727 PMC7066520

[21] Naaz A, Muneshwar KN. How maternal nutritional and mental health affects child health during pregnancy: a narrative review. Cureus. 2023;15(11):e48763. doi:10.7759/cureus.48763 38098932 PMC10719542

[22] Pollock BD, Stuchlik P, Harville EW, . Life course trajectories of cardiovascular risk: impact on atherosclerotic and metabolic indicators. Atherosclerosis. 2019;280:21-27. doi:10.1016/j.atherosclerosis.2018.11.008 30453117 PMC7521150

[23] Krefman AE, Labarthe D, Greenland P, . Influential periods in longitudinal clinical cardiovascular health scores. Am J Epidemiol. 2021;190(11):2384-2394. doi:10.1093/aje/kwab149 34010956 PMC8561125

[24] Ramlakhan KP, Johnson MR, Roos-Hesselink JW. Pregnancy and cardiovascular disease. Nat Rev Cardiol. 2020;17(11):718-731. doi:10.1038/s41569-020-0390-z 32518358

[25] Page KA, Luo S, Wang X, . Children exposed to maternal obesity or gestational diabetes mellitus during early fetal development have hypothalamic alterations that predict future weight gain. Diabetes Care. 2019;42(8):1473-1480. doi:10.2337/dc18-2581 31332028 PMC6647040

[26] Whitaker RC, Pepe MS, Seidel KD, Wright JA, Knopp RH. Gestational diabetes and the risk of offspring obesity. Pediatrics. 1998;101(2):E9. doi:10.1542/peds.101.2.e9 9445519

[27] Gillman MW, Rifas-Shiman S, Berkey CS, Field AE, Colditz GA. Maternal gestational diabetes, birth weight, and adolescent obesity. Pediatrics. 2003;111(3):e221-e226. doi:10.1542/peds.111.3.e221 12612275

[28] Falkegård M, Schirmer H, Løchen ML, Øian P, Acharya G. The validity of self-reported information about hypertensive disorders of pregnancy in a population-based survey: the Tromsø Study. Acta Obstet Gynecol Scand. 2015;94(1):28-34. doi:10.1111/aogs.12514 25250917

[29] Hinkle SN, Rawal S, Zhu Y, Grewal J, Albert PS, Zhang C. Validation of self-reported diagnosis of gestational diabetes at 6-weeks postpartum. Epidemiology. 2017;28(5):747-752. doi:10.1097/EDE.0000000000000695 28570385 PMC5538910

[30] Moth FN, Sebastian TR, Horn J, Rich-Edwards J, Romundstad PR, Åsvold BO. Validity of a selection of pregnancy complications in the Medical Birth Registry of Norway. Acta Obstet Gynecol Scand. 2016;95(5):519-527. doi:10.1111/aogs.12868 26867143

[31] Lloyd-Jones DM, Allen NB, Anderson CAM, ; American Heart Association. Life’s Essential 8: updating and enhancing the American Heart Association’s construct of cardiovascular health: a presidential advisory From the American Heart Association. Circulation. 2022;146(5):e18-e43. doi:10.1161/CIR.0000000000001078 35766027 PMC10503546

[32] Magee LA, Pels A, Helewa M, Rey E, von Dadelszen P; Canadian Hypertensive Disorders of Pregnancy Working Group. Diagnosis, evaluation, and management of the hypertensive disorders of pregnancy: executive summary. J Obstet Gynaecol Can. 2014;36(5):416-441. doi:10.1016/S1701-2163(15)30588-0 24927294

[33] Tsironikos GI, Potamianos P, Zakynthinos GE, Tsolaki V, Tatsioni A, Bargiota A. Effectiveness of lifestyle interventions during pregnancy on preventing gestational diabetes mellitus in high-risk women: a systematic review and meta-analyses of published RCTs. J Clin Med. 2023;12(22):7038. doi:10.3390/jcm12227038 38002654 PMC10672732

[34] Rana S, Lemoine E, Granger JP, Karumanchi SA. Preeclampsia: pathophysiology, challenges, and perspectives. Circ Res. 2019;124(7):1094-1112. doi:10.1161/CIRCRESAHA.118.313276 30920918

[35] McCarthy KJ, Liu SH, Kennedy J, . Preconception HbA1c levels in adolescents and young adults and adverse birth outcomes. JAMA Netw Open. 2024;7(9):e2435136. doi:10.1001/jamanetworkopen.2024.35136 39316396 PMC11423169

[36] Juber NF, Abdulle A, AlJunaibi A, . Maternal early-life risk factors and later gestational diabetes mellitus: a cross-sectional analysis of the UAE Healthy Future Study (UAEHFS). Int J Environ Res Public Health. 2022;19(16):10339. doi:10.3390/ijerph191610339 36011972 PMC9408157

[37] Pedersen DC, Bjerregaard LG, Rasmussen KM, Nohr EA, Baker JL. Risk of gestational diabetes mellitus in nulliparous women - associations with early life body size and change in body mass index from childhood to adulthood. Diabetes Res Clin Pract. 2021;171:108564. doi:10.1016/j.diabres.2020.108564 33271232

[38] Tsviban A, Frenkel A, Schvartz N, Tzur D, Klaitman V, Walfisch A. The association between adolescent obesity and later gestational diabetes in military personnel: A retrospective cohort study. Diabetes Res Clin Pract. 2022;189:109883. doi:10.1016/j.diabres.2022.109883 35504461

[39] Patel SS, Daniels SR. Beginning with the end in mind: the case for primordial and primary cardiovascular prevention in youth. Can J Cardiol. 2020;36(9):1344-1351. doi:10.1016/j.cjca.2020.07.002 32653584

[40] Raitakari O, Pahkala K, Magnussen CG. Prevention of atherosclerosis from childhood. Nat Rev Cardiol. 2022;19(8):543-554. doi:10.1038/s41569-021-00647-9 34987194

[41] Kartiosuo N, Raitakari OT, Juonala M, . Cardiovascular risk factors in childhood and adulthood and cardiovascular disease in middle age. JAMA Netw Open. 2024;7(6):e2418148. doi:10.1001/jamanetworkopen.2024.18148 38913374 PMC11197443

[42] Luirink IK, Wiegman A, Kusters DM, . 20-Year follow-up of statins in children with familial hypercholesterolemia. N Engl J Med. 2019;381(16):1547-1556. doi:10.1056/NEJMoa1816454 31618540

[43] Flanders WD, Lally CA, Zhu BP, Henley SJ, Thun MJ. Lung cancer mortality in relation to age, duration of smoking, and daily cigarette consumption: results from Cancer Prevention Study II. Cancer Res. 2003;63(19):6556-6562. 14559851

[44] Ezzatvar Y, López-Gil JF, Izquierdo M, García-Hermoso A. Maintaining an active lifestyle from adolescence to adulthood might alleviate the adverse association of preterm birth with cardiometabolic health. Diabetes Metab Syndr. 2024;18(2):102966. doi:10.1016/j.dsx.2024.102966 38422778

[45] Dietz P, Bombard J, Mulready-Ward C, . Validation of selected items on the 2003 U.S. standard certificate of live birth: New York City and Vermont. Public Health Rep. 2015;130(1):60-70. doi:10.1177/003335491513000108 25552756 PMC4245286

