# Supplementary material for: Adolescent Cardiovascular Risk Trajectories and Later-Life Maternal Morbidity
Source: JAMA Netw Open. 2025 Oct 10;8(10):e2536095. doi: 10.1001/jamanetworkopen.2025.36095 (PMC12514630; doi:10.1001/jamanetworkopen.2025.36095)
Supplement: Supplement 1. — eFigure 1. Study flowchart eTable 1. Constructed CVH risk score measures in Waves II and III eTable 2. Unweighted counts and weighted frequencies of samples with and without complete data for preconception cardiovascular risk score, age, race/ethnicity, nativity, parental education, insurance status, and wave of first birth, among women without preexisting diabetes and hypertension who gave birth sometime between Waves III to V (birthing sample) eFigure 2. Directed acyclic graph of relationship between adolescent cardiovascular health status (CVH) and later life adverse pregnancy outcomes (APO) eReference [file jamanetwopen-e2536095-s001.pdf]

Supplemental Online Content

McCarthy KJ, Ng A, Boychuk N, Janevic T. Adolescent cardiovascular risk trajectories and later maternal morbidity. *JAMA Netw Open*. 2025;(8):e2536095. doi: 10.1001/jamanetworkopen.2025.36095

eFigure 1. Study flowchart

eTable 1. Constructed CVH risk score measures in Waves II and III

eTable 2. Unweighted counts and weighted frequencies of samples with and without complete data for preconception cardiovascular risk score, age, race/ethnicity, nativity, parental education, insurance status, and wave of first birth, among women without preexisting diabetes and hypertension who gave birth sometime between Waves III to V (birthing sample)

eFigure 2. Directed acyclic graph of relationship between adolescent cardiovascular health status (CVH) and later life adverse pregnancy outcomes (APO)

eReference

This supplemental material has been provided by the authors to give readers additional information about their work.

eFigure 1. Study flowchart.

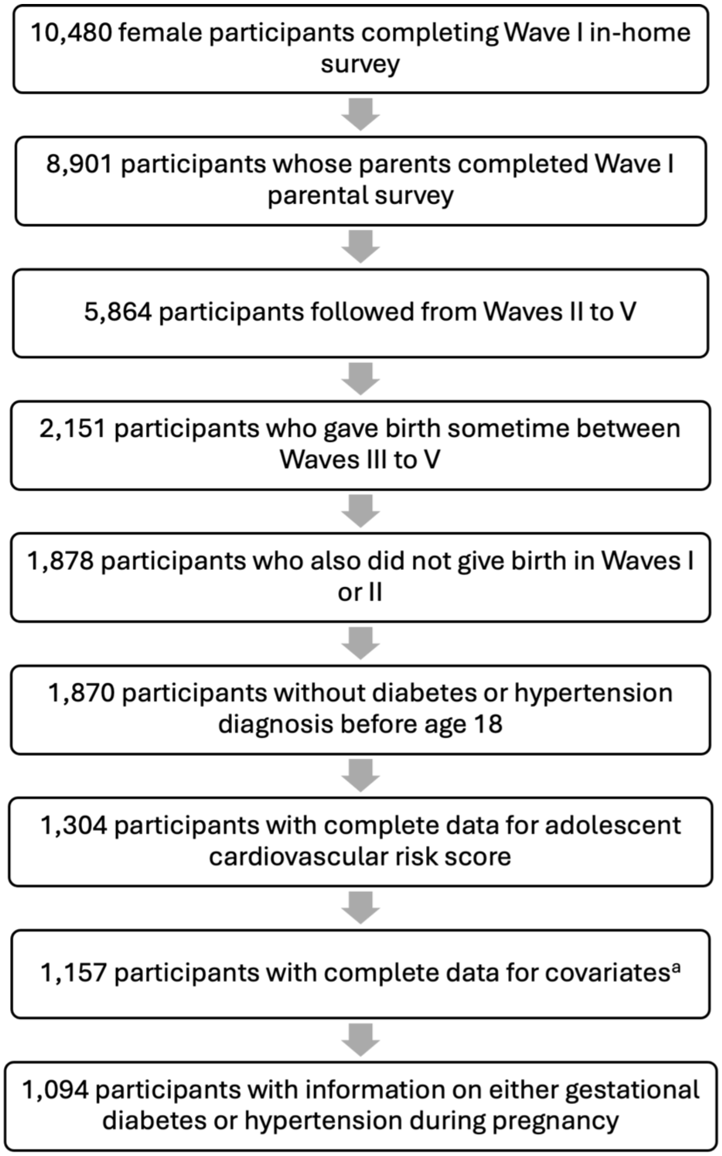

[a]age, race and ethnicity, parental education, insurance status, and wave of first birth.

© 2025 McCarthy KJ et al. *JAMA Network Open*.

eTable 1. Constructed CVH risk score measures in Waves II and III.

Metric	LE8 Scoring for Adolescents[1]	Adolescent (Wave II)	LE8 Scoring for Adults[1]	Young Adult (Wave III)
**Diet**	**Self-reported daily intake of a DASH-style eating pattern**	**Self-reported fast food consumption[a] in the last seven days**	**Self-reported daily intake of a DASH-style eating pattern**	**Self-reported fast food consumption[a] in the last seven days**
	100: ≥ 95th percentile (ideal diet)	100: 0 days	100: ≥ 95th percentile (ideal diet)	100: 0 days
	80: 75th-94th percentile	80: 1-2 days	80: 75th-94th percentile	80: 1-2 days
	50: 50th-74th percentile	50: 3-4 days	50: 50th-74th percentile	50: 3-4 days
	25: 25th-49th percentile	25: 5-6 days	25: 25th-49th percentile	25: 5-6 days
	0: 1st-24th percentile (least ideal)	0: 7 days	0: 1st-24th percentile (least ideal)	0: 7 days
**Physical Activity**	**Self-reported minutes of moderate- (or greater) intensity activity per week**	**Self-reported sweat or breathe heavily from work, play, exercise in a normal week**	**Self-reported minutes of moderate- (or greater) intensity activity per week**	**Self-reported number of times engaged in physical activities[b] in the last seven days**
	100: ≥ 420	100: >7 times per week	100: ≥ 150	100: >9 times
	90: 360-419<br>80: 300-359	75: 6-7 times per week	90: 120-149<br>80: 90-119	75: 5-8 times
	60: 240-299<br>40: 120-239	50: 3-5 times per week	60: 60-89<br>40: 30-59	50: 2-4 times
	20: 1-119	25: 1-2 times per week	20: 1-29	25: 1 time
	0: 0	0: 0 times per week	0: 0	0: 0 times
**Nicotine Exposure**	**Self-reported use of cigarettes or inhaled nicotine delivery system**	**Self-reported cigarette use[c] and exposure to household secondhand smoke**	**Self-reported use of cigarettes or inhaled nicotine delivery system**	**Self-reported cigarette use[c]**
	100: Never tried	100: Never smoker	100: Never smoker	100: Never smoker
	50: Tried any nicotine product, but >30 days ago	50: Ever smoked, but more than 30 days ago	75: Former smoker, quit ≥5 yrs	50: Former smoker, didn't smoke in Wave

© 2025 McCarthy KJ et al. *JAMA Network Open*.

			50: Former smoker, quit 1-<5 yrs	II and not currently smoking
	25: Currently using inhaled nicotine delivery system		25: Former smoker, quit <1 yr, or currently using inhaled NDS	25: Former smoker, smoked at Wave II and not currently smoking
	0: Current combustible use within past 30 days	0: Current smoker	0: Current smoker	0: Current smoker
	Subtract 20 points (unless score is 0) for living with active indoor smoker at home	Subtract 20 points (unless score is 0) for living with cigarette smoker at home	Subtract 20 points (unless score is 0) for living with active indoor smoker in home	Data on cigarette smoker at home not collected
**Sleep health**	**Self-reported average hours of sleep per night**	**Self-reported average hours of sleep per night[d]**	**Self-reported average hours of sleep per night**	**Self-reported sleep duration[d]**
	100: 8-10 hours (age-appropriate optimal range)	100: 8-10 hours	100: 7 - < 9 hours	100: 7 - < 9 hours
	90: < 1 hour above optimal		90: 9 - < 10 hours	90: 9 - < 10 hours
	70: > 1 hour below optimal		70: 6 - < 7 hours	70: 6 - < 7 hours
	40: 1 - < 2 hours below or ≥ 2 hours above optimal	40: 7 hours or ≥ 11 hours	40: 5 - < 6 or ≥ 10 hours	40: 5 - < 6 or ≥ 10 hours
	20: 2 - < 3 hours below optimal	20: 6 hours	20: 4 - < 5 hours	20: 4 - < 5 hours
	0: ≥ 3 hours below optimal	0: 5 hours or lower	0: < 4 hours	0: < 4 hours
**Body mass index (BMI)**	**BMI percentiles for age and sex**	**BMI calculated from self-reported height and weight (categorized into percentiles for age and sex if ≤ 19 years)**	**BMI (kg/m^2)**	**BMI (kg/m^2) calculated from self-reported height and weight (categorized into percentiles for age and sex if ≤ 19 years)**
	100: 5th - < 85th percentile	100: 5th - < 85th percentile	100: < 25	100: < 25
	70: 85th - < 95th percentile	70: 85th - < 95th percentile	70: 25.0-29.9	70: 25.0-29.9

© 2025 McCarthy KJ et al. *JAMA Network Open*.

	30: 95th - < 120th of the 95th percentile	30: 95th - < 120th of the 95th percentile	30: 30.0-34.9	30: 30.0-34.9
	15: 120% of the 95th percentile - <140% of the 95th percentile	15: 120% of the 95th percentile - <140% of the 95th percentile	15: 35.0-39.9	15: 35.0-39.9
	0: ≥ 140% of the 95th percentile	0: ≥ 140% of the 95th percentile	0: ≥ 40.0	0: ≥ 40.0
**Blood lipids**	**Non-HDL cholesterol (mg/dL)**	**Biomarker data unavailable**	**Non-HDL cholesterol (mg/dL)**	**Biomarker data unavailable**
**Blood glucose**	**FBG (mg/dL) or HbA1c (%)**	**Biomarker data unavailable**	**FBG (mg/dL) or HbA1c (%)**	**Biomarker data unavailable**
**Blood pressure**	**Systolic and diastolic (mm Hg)**	**Biomarker data unavailable**	**Systolic and diastolic (mm Hg)**	**Biomarker data unavailable**

[a] Responses to the questionnaire item, "In the last seven days, on how many days did you eat at a fast food type place—McDonalds, Kentucky Fried Chicken, Pizza Hut, Taco Bell, etc.?" Fast food consumption was used as a proxy for DASH (Dietary Approaches to Stop Hypertension) dietary adherence due to limited survey questions about nutrient intake.

[b]Information on minutes of physical activity per week was not available. Scoring was based on distribution of the sum of the following activities: (1) Bike/skateboard/dance/hunt/do yard work, (2) rollerblade/roller skate/downhill ski/snowboard/play racquet sports/do aerobics, (3) participate in strenuous team sports such as football/soccer/basketball/lacrosse/rugby/field hockey/ice hockey, (4) play individual sport, (5) participate in gymnastics/weight lifting/strength training, (6) walk for exercise.

[c] Nicotine exposure calculated from response to following items: "Have you ever tried cigarette smoking, even just 1 or 2 puffs?", "Have you ever smoked cigarettes regularly, that is, at least 1 cigarette every day for 30 days?", and "During the past 30 days, on how many days did you smoke cigarettes?" In Wave I, cigarette smokers in household were self-reported by parents. Information on inhaled nicotine delivery system use was not available.

[d] In Wave II, response to "How many hours of sleep do you usually get?" Scoring was modified because survey responses were categorized as whole hours. In Wave III, calculated using responses to "On days when you go to work , school, or similar activities, what time do you usually wake up?" and "On days when you go to work school, or similar activities, what time do you usually go to sleep the night (or day) before?"

© 2025 McCarthy KJ et al. *JAMA Network Open*.

eTable 2. Unweighted counts and weighted frequencies of samples with and without complete data for preconception cardiovascular risk score, age, race/ethnicity, nativity, parental education, insurance status, and wave of first birth, among women without preexisting diabetes and hypertension who gave birth sometime between Waves III to V (birthing sample).

	Birthing Sample (N=2083)	Birthing Sample with Complete Data (N=1094)
Median age (IQR)	16 (15-17)	16 (14-17)
Median BMI (IQR)	21.7 (19.6-24.6)	21.6 (19.6-24.5)
Median CVH score, Wave II (IQR)	66 (54-76)	66 (54-76)
Median CVH score, Wave III (IQR)	66 (54-78)	66 (54-78)
**Age group**	*No. (%)*	*No. (%)*
12 to 14 years	422 (24.5)	204 (25.6)
15 to 17 years	1294 (61.1)	689 (59.4)
18 to 22 years	367 (14.4)	201 (15.0)
**Race/ethnicity**		
Asian	64 (1.9)	31 (1.3)
Black	420 (14.3)	200 (13.6)
Hispanic	278 (10.2)	123 (8.0)
Other	149 (6.7)	76 (6.7)
White	1169 (66.8)	664 (70.4)
Missing	3	0
**Nativity**		
US born	1965 (95.7)	1045 (96.9)
Foreign born	118 (4.3)	49 (3.1)
**Parental Education**		
Less than high school	327 (17.6)	169 (15.6)
High school completion	618 (36.2)	375 (37.8)
Some college	564 (30.9)	334 (30.0)
College degree or higher	346 (15.3)	216 (16.6)
Missing	228	0
**Insurance**		
Public or none	638 (38.4)	352 (37.2)
Private insurance	1207 (61.6)	742 (62.8)
Missing	238	0
**Median Wave of First Birth (IQR)**	3 (3-4)	3 (3-4)
**HDP**		
No	1498 (76.0)	825 (75.5)
Yes	468 (24.0)	269 (24.5)
Missing	117	0

© 2025 McCarthy KJ et al. *JAMA Network Open*.

**Gestational Hypertension**		
No	1605 (81.9)	882 (81.4)
Yes	353 (18.1)	209 (18.6)
Missing	125	3
**Preeclampsia or Eclampsia**		
No	1646 (83.6)	915 (83.4)
Yes	312 (16.4)	176 (16.6)
Missing	125	3
**GDM**		
No	1754 (90.6)	969 (90.6)
Yes	199 (9.4)	121 (9.4)
Missing	130	4

CVH=cardiovascular health. HDP=hypertensive disorders of pregnancy. GDM=gestational diabetes.

Birthing sample: n=68 missing BMI, n=636 missing wave II score, n=568 missing wave III score

Complete data sample: n=192 missing wave III score

© 2025 McCarthy KJ et al. *JAMA Network Open*.

eFigure 2. Directed acyclic graph of relationship between adolescent cardiovascular health status (CVH) and later life adverse pregnancy outcomes (APO).

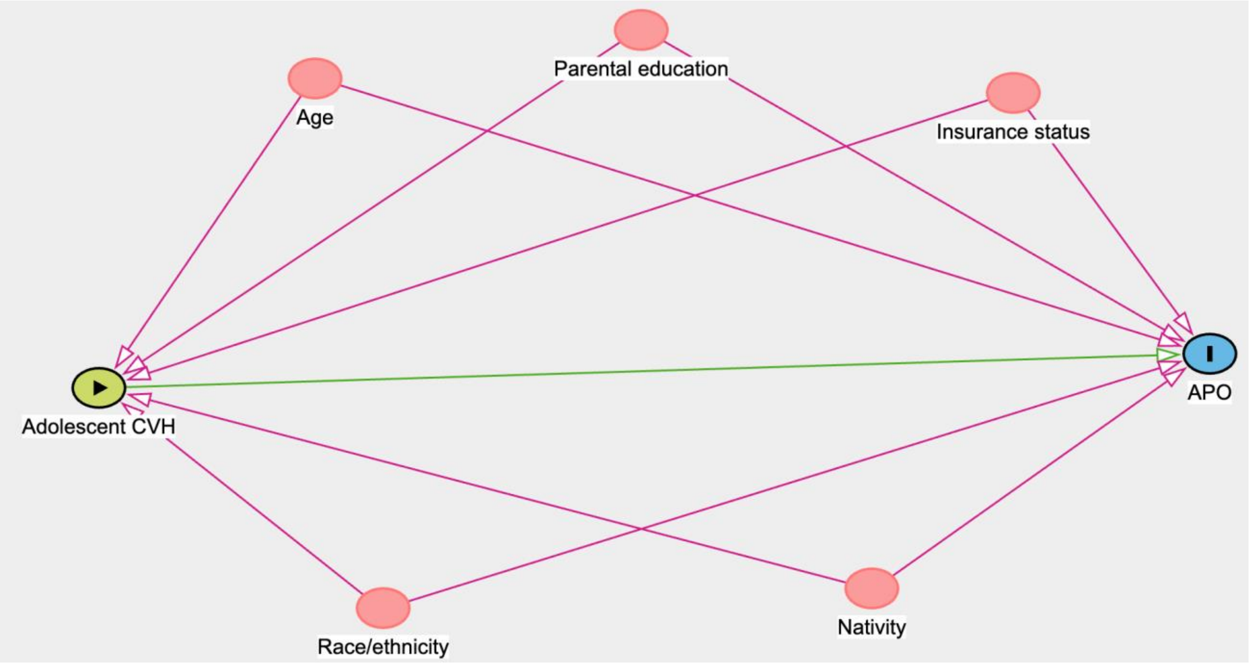

eReference

1. Lloyd-Jones DM, Allen NB, Anderson CAM, et al. Life's Essential 8: Updating and Enhancing the American Heart Association's Construct of Cardiovascular Health: A Presidential Advisory From the American Heart Association. *Circulation*. 2022;146(5). doi:10.1161/CIR.0000000000001078

© 2025 McCarthy KJ et al. *JAMA Network Open*.
