# Supplementary material for: Adolescent Cardiovascular Risk Trajectories and Later-Life Maternal Morbidity
Source: JAMA Netw Open. 2025 Oct 10;8(10):e2536095. doi: 10.1001/jamanetworkopen.2025.36095 (PMC12514630; doi:10.1001/jamanetworkopen.2025.36095)
Supplement: Supplement 2. — Data Sharing Statement [file jamanetwopen-e2536095-s002.pdf]

Data Sharing Statement

McCarthy. Adolescent Cardiovascular Risk Trajectories and Later Life Maternal Morbidity. *JAMA Netw Open*. Published October 07, 2025. doi:10.1001/jamanetworkopen.2025.36095

Data
Data available: No

Additional Information
Explanation for why data not available: Restricted use data from the National Longitudinal Adolescent and Adult Health Study is available upon application to the Carolina Population Center.
